# Diversity and Distribution Patterns of Cetaceans in the Subtropical Southwestern Atlantic Outer Continental Shelf and Slope

**DOI:** 10.1371/journal.pone.0155841

**Published:** 2016-05-31

**Authors:** Juliana Couto Di Tullio, Tiago B. R. Gandra, Alexandre N. Zerbini, Eduardo R. Secchi

**Affiliations:** 1 Laboratório de Ecologia e Conservação da Megafauna Marinha (ECOMEGA), Instituto de Oceanografia, Universidade Federal do Rio Grande, Rio Grande, Rio Grande do Sul, Brasil; 2 Laboratório de Geotecnologias e Meio Ambiente (GEOMA), Instituto Federal de Educação, Ciência e Tecnologia do Rio Grande do Sul (IFRS), Rio Grande, Rio Grande do Sul, Brasil; 3 Cascadia Research Collective, Olympia, Washington, United States of America; 4 National Marine Mammal Laboratory, Alaska Fisheries Science Center, National Marine Fisheries Service, National Oceanic and Atmospheric Administration, Seattle, Washington, United States of America; 5 Instituto Aqualie, R. Dr. Paulo Japiassú Coelho 714/206, Juiz de Fora, MG, Brazil; Hellenic Centre for Marine Research, GREECE

## Abstract

Temporal and spatial patterns of cetacean diversity and distribution were investigated through eight ship-based surveys carried out during spring and autumn between 2009 and 2014 on the outer continental shelf (~150m) and slope (1500m) off southeastern and southern Brazil (~23°S to ~34°S). The survey area was divided into southeast and south areas according to their oceanographic characteristics. Twenty-one species were observed in 503 sightings. The overall number of species was similar between the two areas, though it was higher in the spring in the south area. Five species were dominant and diversity varied more seasonally than spatially. ANOVA and kernel analyses showed that overall cetacean densities were higher in spring compared to autumn. *Physeter macrocephalus*, the most frequent species, concentrated throughout the south area at depths over 1000m in both seasons. Despite the overlapped occurrence at a broader scale, small delphinids presented latitudinal and in-offshore gradients as well as seasonal variation in distribution patterns, which could indicate habitat partitioning between some species. *Delphinus delphis* was only recorded in the south and its density decreased in areas where the presence of *Stenella frontalis* increased, mainly beyond the 250m isobath. Densities of *S*. *longirostris* and *S*. *attenuata* increased in lower latitudes and beyond the shelf break. The large delphinids *Tursiops truncatus* and *Globicephala melas* formed mixed groups in many occasions and were observed along the study area around depths of 500m. *Grampus griseus* was twice as frequent in the south area and densities increased in waters deeper than 600m. As expected, densities of both small and large migratory whales were higher during spring, over the continental slope, in the southeast area. The results presented here provided strong evidence on the importance of the outer continental shelf and slope to a diverse community of cetaceans occurring in the subtropical Southwestern Atlantic.

## Introduction

Oceanic productive areas are known to aggregate high species richness and abundance of top predators, such as cetaceans, and are usually situated near hydrographic fronts and abrupt topographies which are characterized by strong sea surface temperature gradients and high chlorophyll concentrations [1,2,3]. In southern Australia, for example, the most common cetacean species were associated to upwelling season and migration cycles [4]. In the oceanic waters of Gulf of Mexico species appeared to concentrate near the slope or around eddies, where the amount of potential prey for cetaceans may be consistently greater in some seasons [5,6]. Six of the most common cetaceans recorded off Southern California had seasonal different spatial distribution and abundance oscillation which can be related to water masses, depth and *El Niño* and *La Niña* events [7]. Hence, habitat features such as depth, slope, distance from oceanographic processes (*e*.*g*. upwelling) that enhance local productivity and prey aggregations are key factors to determine patterns of cetacean distribution [5, 8, 9, 10].

There is a high diversity of cetaceans along the continental shelf and slope off Brazilian waters, including species associated with tropical and temperate waters as well as those considered cosmopolitan [11,12,13]. However, most information on cetacean distribution in Brazilian offshore waters comes from the national observers programs of the oil and gas industry (*e*.*g*. [14]), which lacks standardized procedures, sampling methods and experienced researchers. A few research expeditions made by experienced researchers to collect data on cetacean distribution in offshore waters of southern and southeastern Brazil were opportunistic and restricted in space and time [11, 15], therefore the richness of cetacean species recorded in those surveys is probably underestimated. In fact, stranding records along the southeastern and southern Brazilian coasts suggest that a much higher number of cetacean species may occur in offshore waters of these regions [16,17,18]. Furthermore, spatial and seasonal variation in distribution and occurrence patterns has yet to be determined.

The Brazilian continental shelf and slope is an economically important region for fisheries and the oil and gas industries. Approximately 60% of the national commercial fish catch come from highly productive waters of the southeastern and southern continental shelf and slope [19]. These two regions are characterized by different dominant hydrographic dynamics. The southeastern continental shelf and slope is mostly influenced by the tropical waters of the Brazil current, which transports tropical warm and oligotrophic water. In this area, higher productive zones are occasionally triggered by upwellings that pump the South Atlantic Central Water to superficial layers, changing composition and enhancing densities of phytoplankton communities [20]. During winter, the upwellings are associated with cyclonic meanders of Brazil current and in summer by a combination of the steep topography of the shelf break and wind driven upwelling [21,22]. On the other hand, the southern continental shelf and slope are under the influence of the Brazil current, the sub-Antarctic waters transported by the Malvinas/Falkland currents (M/FC) and continental waters from La Plata River and Patos Lagoon plumes, which form the subtropical shelf front (STSF) [23,24]. The STSF is characterized by a sharp thermohaline transition between these water masses. This front changes its intensity and location over the continental shelf according to predominance of southerly and northerly wind seasonal regimes and the continental water is exported towards the shelf break and slope [24, 25]. These processes and surface and subsurface upwelling events influence the productivity in offshore waters by enhancing concentration of inorganic nutrients, chlorophyll-a (Chl-a) and density of zooplankton [26, 27].

A global meta-analysis on diversity and predictors of cetacean occurrence suggests that a larger number of species are expected to inhabit the subtropical waters of southeastern and southern Brazil compared to tropical waters Brazil [2]. Since oceanographic processes and productivity in the outer continental shelf and slope vary seasonally and the southern region is under the influence of a variety of water masses, it is anticipated that richness and seasonal variations in cetacean diversity is higher in the southern region. It is also expected that the density of cetaceans is higher at closer proximity to the shelf break, since subsurface processes that enhance productivity predominate in this region. The present study aimed at assessing spatial and seasonal (spring and autumn) distribution patterns and diversity of cetacean in the outer continental shelf and slope off southern and southeastern Brazil. Our results showed that less than five species presented relative abundance above 10% of overall cetacean, and that diversity varies more seasonally than spatially. Furthermore, we provided a comprehensive description of the spatial and seasonal distribution patterns of the most frequent species inhabiting this area during the austral spring and autumn seasons. This information can be useful for identifying biological and ecological significant areas for conservation and, thus, to regulate intense fishing and oil exploration activities according to the occurrence patterns of the cetacean species.

## Materials and Methods

### Survey design and data collection

**Fig 1:**
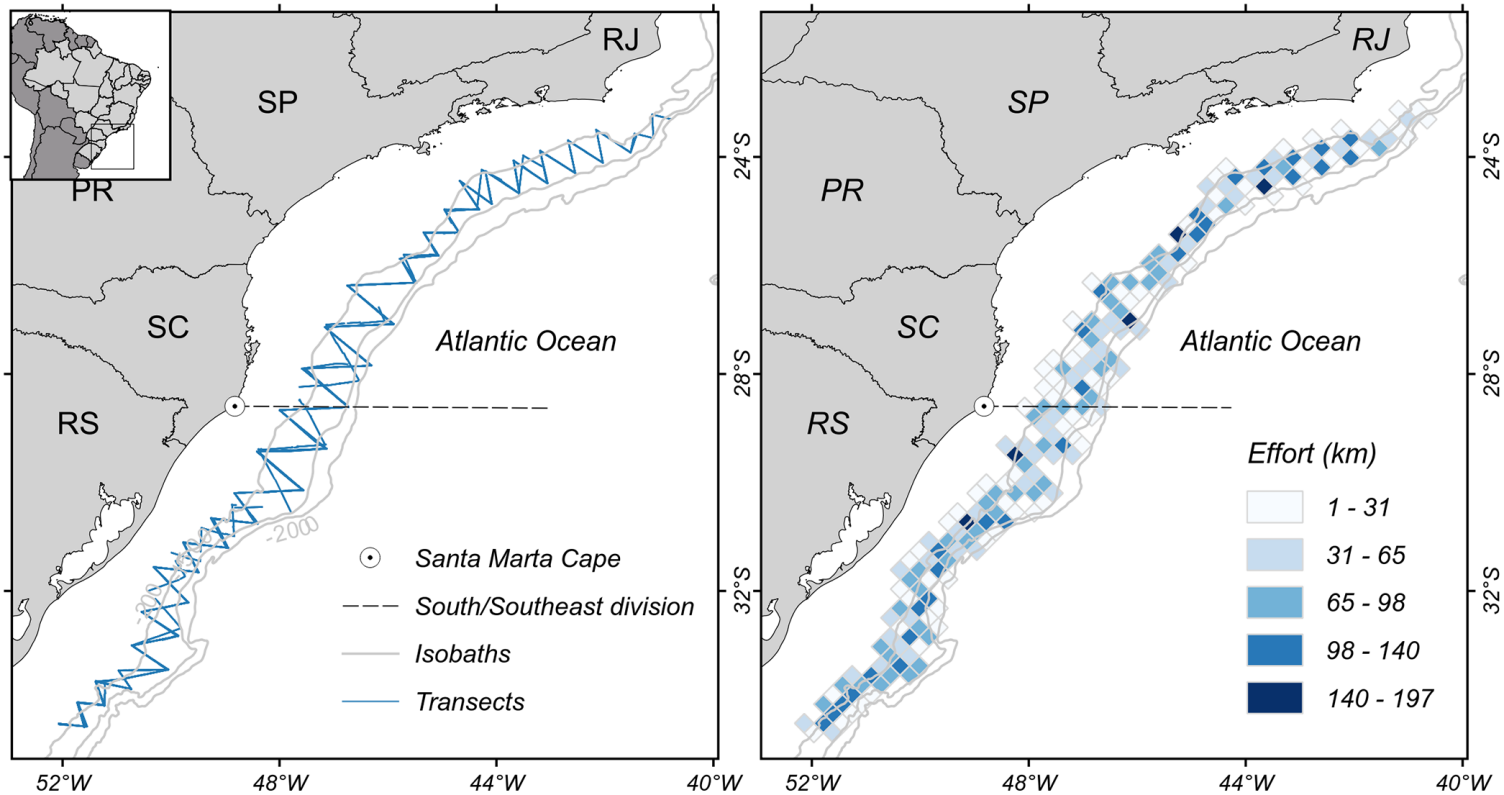
Ship-based cetacean survey tracks and effort in outer continental shelf and slope off southern and southeastern Brazil between 2009–2014. A) Zig-zag transects lines followed by the ship. B) Grid cells are 0.25°x0.25° and darker shading indicates greater searching effort. Acronyms represent the Brazilian states of Rio Grande do Sul (RS); Santa Catarina (SC); Paraná (PR); São Paulo (SP) and Rio de Janeiro (RJ).

Eight surveys were conducted during austral spring (n = 4) and autumn (n = 4) between 2009 and 2014 onboard the 36 meter-long R/V *Atlântico Sul* of the Federal University of Rio Grande (FURG) following approximately the same transect lines (Fig 1). Zig-zag transect lines were pre-designed to cover the outer continental shelf and slope of southeastern (22.9°S) and southern (33.7°S) Brazil, from approximately the 150 to the 1500m isobaths (Fig 1). For logistic reasons, the surveys started at the southernmost transect line. The vessel’s steering speed varied between 14.4–18.5 km/h (8–10kt). Due to weather conditions or ship schedule, the surveys started at different dates and effort varied along the study area (Table 1).

**Table 1:** Summary of the survey effort (km) for each year and season in south and southeast Brazil between 2009 and 2014.

		Area				
Survey (year)	Season	South	Southeast	Total (km)	Starting date (day/month)	N days
1 (2009)	Spring	1267.6	1028.2	2295.7	22/10	15
2 (2010)	Autumn	1688.8	488.6	2177.4	22/04	22
3 (2010)	Spring	1543.0	1969.5	3512.5	20/10	31
4 (2011)	Autumn	1786.8	1678.2	3465.1	13/04	29
5 (2012)	Spring	1827.6	1443.9	3271.5	26/10	29
6 (2013)	Autumn	1786.5	1347.6	3134.1	07/05	34
7 (2014)	Autumn	1375.4	925.2	2300.6	10/05	30
8 (2014)	Spring	1604.6	881.7	2486.3	12/11	30
Total		12880.4	9762.8	22643.2		220

Starting date, day which the survey started; N days, duration of each survey in days. Effort considered only with sea state ≤ 5 of Beaufort scale

Two researchers (henceforth referred as observers) searched for cetacean from the flying bridge (observation height ~ 8.6m). The observers, positioned in the port and starboard sides, searched for cetacean by scanning from the vessel’s bow to 90° of their side. During the searching procedure, the observers alternated using Fujinon 7x50 reticule binoculars and with unaided eyes. A third researcher (henceforth referred as assistant) was positioned behind the observers to help species identification and group size estimates after detection. The main role of this assistant was to minimize the time that the observers take to resume scanning after detection. A fourth researcher was in charge to record the data in a notebook connected to the vessel’s navigation system using program WinCruz (available at: http://swfsc.noaa.gov/textblock.aspx?Division=PRD&ParentMenuId=147&id=1446). The information recorded included effort *(e*.*g*. date, time, coordinates), sighting conditions (*e*.*g*. sea stated in Beaufort scale), sighting data (*e*.*g*. species, number of individuals, and position based on the radial distance—calculated from the binocular’s reticles—and angle relative to the ship’s heading—measured using an angle board) and observers’ position. Six to eight trained observers rotated though the observation positions every 30 minutes. Group size was estimated by consensus between the observer and the assistant. Best, low and high estimates of group size were recorded, though only “best” was used in the analyses. Cetacean were identified to the lowest possible taxonomic level and attributed to generic categories (*e*.*g*. large whale, small dolphin) for some analyses.

Most of the time transects were surveyed using passing mode in which species and group size were determined without the vessel diverting from the trackline (*e*.*g*. [28]). In a few occasions, however, effort was halted and the ship closed in the sighting, for no longer than one hour, in order to identify the species and/or to better estimate group size. This happened only when the detected group had passed abeam and no other group had been seen. After species identification and/or group size estimation the effort was resumed at the location where it ended. In order to standardize sighting effort, only sightings made by the two observers and at sea state 5 or lower (Beaufort scale) were considered in the analyses.

### Analyses

The study area was divided into areas north (herein referred as southeast) and south of Santa Marta Grande Cape (28.6°S) (Fig 1) on the basis of distinct oceanographic characteristics influencing these areas [19]. The southeast area is predominantly influenced by the oligotrophic waters of Brazil Current and higher productive zones are occasionally triggered by upwellings [22]. In the south area, the Brazil current, upwellings and seasonal influences of rich coastal water discharge enhances the productivity [20,24]. Effort was measured (in kilometres) as the distance travelled on transects for each area and season.

#### Species Richness and Diversity

Species diversity between areas and seasons was measured using beta-diversity index of Whittaker with Harrison et al.’s modification to account for differences in sampling effort [29]. Based on four criteria (turnover rate, additivity, independence of alpha-diversity, independence of excessive sample size), this index performed better against other five indexes [30, 31]. The Whittaker plot was also used to visually represent species richness (S, the number of species) and evenness. This plot considers the relative abundance as a proportion of the total number of individuals of each species in relation to the total number of individuals of all species [31]. The similarity (1-β diversity) between areas and seasons was evaluated using the index of Morisita-Horn as it is less influenced by species richness and sample size. Given that this index is sensitive to species abundance, the number of individuals of each sighting was square-rooted to minimize this effect [31, 32, 33]. The beta-diversity indices were calculated using vegdist and ht functions in vegan and MBI packages, respectively [34, 35] in R version 3.1.2 [36].

#### Spatial and Temporal Variation in Distribution Patterns

Encounter rate (ER), as the number of individuals per distance surveyed on effort (ind/km), was assumed to represent density and was used to investigate spatial (south versus southeast) and temporal (spring versus autumn) variation in distribution patterns of cetacean in the study area. In order to overcome small sample size issues (low sighting frequency for some species), sightings were pooled into four group categories according to body size or likely ecological niche: small delphinids, medium-large delphinids (herein referred as large delphinids), small-medium whales (herein referred as small whales) and large whales (Table 2). Due to the high sighting frequency of sperm whale (*Physeter macrocephalus*) and its particular niche (*i*.*e*. deep-water squid feeder), this species was not grouped in any of those categories. Because only one beaked whale (Ziphiidae) was sighted (Table 2), this record was excluded from the analyses. Encounter rate values were square-rooted through box-cox power transformation to maximise normality and homoscedasticity [37]. Spatial and temporal variations in ER, calculated by survey, were assessed through ANOVA followed by post-Hoc Tukey tests for both group categories and individual species when the number of sightings was higher than 15. The normality and variance of the ANOVA’s residuals were investigated with Shapiro-Wilk and Levene’s test [38]. Analyses were performed using the MASS and car packages in R version 3.1.2 [36, 39, 40].

**Table 2:** Species richness and summary of cetacean sightings distribution considering species, group categories, seasons, areas and depth.

		South Area					Southeast Area					Total Study Area	
Species group categories	Scientific names	Spring (S = 15)		Autumn (S = 9)		Total (S = 16)	Spring (S = 14)		Autumn (S = 14)		Total (S = 17)	Total (S = 21)	Depth (m)
		N	NIMean (se)	N	NIMean (se)	NIMean (se)	N	NIMean (se)	N	NIMean (se)	NIMean (se)	NIMean (se)	Depth Mean (se)
Small delphinids	*Delphinus delphis*	22	136.68 (65.22)	5	214.8 (139.86)	151.15 (58.24)	0	0	0	0	0	151.15 (58.24)	243.2 (14.2)
	*Feresa attenuata*	0	0	0	0	0	1	10	0	0	0	10	834
	*Stenella attenuata*	0	0	0	0	0	6	117.5 (48.88)	1	25	104.29 (43.38)	104.29 (43.38)	755.7 (19.2)
	*Stenella clymene*	2	178 (172)	0	0	178 (172)	1	10	0	0	10	122 (114.1)	1171 (14.8)
	*Stenella coeruleoalba*	2	110 (10)	0	0	0	0	0	0	0	0	110 (10)	1299 (13.6)
	*Stenella frontalis*	15	205.07 (69.02)	10	25.1 (6.88)	133 (44.7)	10	189.1 (66.19)	5	66.4 (42.56)	148.2 (47.85)	138.75 (32.83)	397.5 (16.1)
	*Stenella longirostris*	2	495 (405)	0	0	495 (405)	11	258.18 (52.98)	2	450 (350)	287.69 (62.84)	315.33 (69.64)	692.5 (17.6)
	*Steno bredanensis*	1	20	0	0	0	0	0	0	0	0	20	145
	Unidentified Dolphin	7	4.85 (1.4)	2	8.5 (1.5)	5.67 (1.22)	4	1.75 (0.25)	4	2.5 (0.65)	2.13 (0.35)	4 (0.79)	*
Large delphinids	*Globicephala melas*	10	121.5 (56.81)	5	80 (30.49)	107.46 (38.71)	1	35	1	15	25 (10)	97.76 (34.67)	637.9 (18.4)
	*Globicephala* spp	0	0	0	0	0	0	0	0	0	0	3	1223
	*Grampus griseus*	3	30.67 (13.37)	3	23.33 (10.14)	27 (7.68)	1	13	2	265 (35)	181 (86.39)	78.33 (108.39)	1095.8 (16.3)
	*Orcinus orca*	3	9 (3.79)	0	0	9 (3.79)	0	0	2	6.5 (1.5)	6.5 (1.5)	8 (4.95)	550.9 (22.1)
	*Pseudorca crassidens*	0	0	0	0	0	1	40	1	30	35 (5)	35 (5)	705.7 (25.5)
	*Tursiops truncatus*	6	47.33 (31.29)	9	64.89 (18.43)	57.87 (16.17)	12	19.75 (5.68)	6	17 (6.81)	18.83 (4.31)	36.58 (8.32)	575.3 (19.3)
Small whales	*Balaenoptera acutorostrata*	3	1.33 (0.33)	1	1	1.25 (0.25)	1	3	2	2 (1)	2.33 (0.67)	1.72 (0.36)	553.7 (19.9)
	*Balaenoptera bonaerensis*	1	2	1	2	2	0	0	1	8	8	4 (2)	410.7 (17.2)
	*Balaenoptera* spp (like minke whale)	4	1.5 (0.5)	1	1	1.4 (0.4)	8	1.37 (0.18)	2	1	1.3 (0.15)	1.3 (0.16)	635.9 (19.6)
	Unidentified small whale	2	1	1	1	1	2	1	0	0	0	1	*
Large whales	*Balaenoptera borealis*	1	1	0	0	1	0	0	0	0	0	1	471
	*Balaenoptera brydei*	1	1	0	0	1	9	1.56 (0.18)	4	1.25 (0.25)	1.46 (0.14)	1.42 (0.14)	755.2 (18.6)
	*Balaenoptera physalus*	0	0	1	1	1	0	0	2	6	6	4.33 (1.67)	585.8 (11.7)
	*Megaptera novaeangliae*	0	0	0	0	0	11	2.36 (0.43)	3	1 (0)	2.07 (1.38)	2.07 (1.38)	560.2 (21.6)
	*Balaenoptera* spp.	1	2	4	1.5 (0.29)	1.6 (0.24)	2	1	4	1.25 (0.25)	1.16 (0.17)	1.36 (0.15)	*
	Unidentified large cetacean	41	1.46 (0.16)	6	1.17 (0.17)	1.42 (0.14)	32	1.06 (0.04)	14	1.07 (0.07)	1.07 (0.04)	1.25 (0.08)	*
	Unidentified large whale	5	1.2 (0.2)	3	1.3 (0.33)	1.25 (0.16)	3	1 (0)	6	1.16 (0.17)	1.1 (0.11)	1.18 (0.09)	*
Sperm Whale	*Physeter macrocephalus*	86	3.35 (0.37)	27	7.56 (3.99)	4.35 (0.99)	19	2.26 (0.37)	8	15.75 (12.07)	6.26 (3.62)	4.72 (1.06)	1223.6 (19.8)
Unidentified Ziphiidae Whale	ziphiid whale	0	0	0	0	0	1	1	0	0	1	1	601

S, species richness is the number of species identified. Total spring richness was 20 and total autumn richness 15.

N, number of sightings; NI Mean, mean number of individuals; se, standard error.

* depths of unidentified species were not considered.

Grid cells of 0.25 x 0.25 degrees were generated in maps built in QGIS 12.12 –Lyon (http://qgis.org) and used to represent the distribution patterns of cetacean group categories. For each cell ER values were summarized by season as ER_ij_ = Σn_i_*10/Σeff_j_ where: n_i_ is the number of individuals of species i, j is the cell, eff is the distance travelled, and was calculated through spatial and temporal functions using PostgreSQL 9.3 and PostGIS 2.1 [41]. Kernel density plots were used to investigate the seasonal patterns of cetacean species distribution in regard to latitude and depth weighted by ER for each cell. Plots were also produced for species with small number of sightings (> 4, the least number of events necessary for the kernel plot) in order to provide a preliminary view on the distribution of infrequent species in this region. The kernel density bandwidth was determined using SJ method [42]. The plots were made using stat_density function in ggplot2 package [43] in R version 3.1.2 [36].

## Results

Effort varied between surveys from 2177.4km to 3512.5km, mainly due to weather conditions, and was higher during spring and in the south area (Tables 1 and 3), which was more evenly covered between seasons (Table 3, Fig 1).

**Table 3:** Differences in survey effort tested between areas and seasons using Chi-square test (X^2^).

	X^2^	p-value
Total effort between seasons	8.4	**0.0038**
Total effort between areas	93.1	**0.0001**
During spring between areas	8.5	**0.0037**
During autumn between areas	116.8	**0.0001**
South Area: between seasons	3.13	0.0784
Southeast Area: between seasons	37.642	**0.0001**

X^2^, values of the chi-square test; p-value, values <0.05 were considered significant.

A total of 503 sightings were recorded during this study. Identification to species level (for a total of 21 species) was possible for 344 sightings (Table 2). Cetaceans were identified to genus level or were classified as unidentified in 26 and 133 occasions, respectively. Baleen whales were represented by six species of the family Balaenopteridae. Toothed cetaceans were represented by fourteen species in the Delphinidae, Physeteridae and Ziphiidae families.

### Species Richness and Diversity

**Fig 2:**
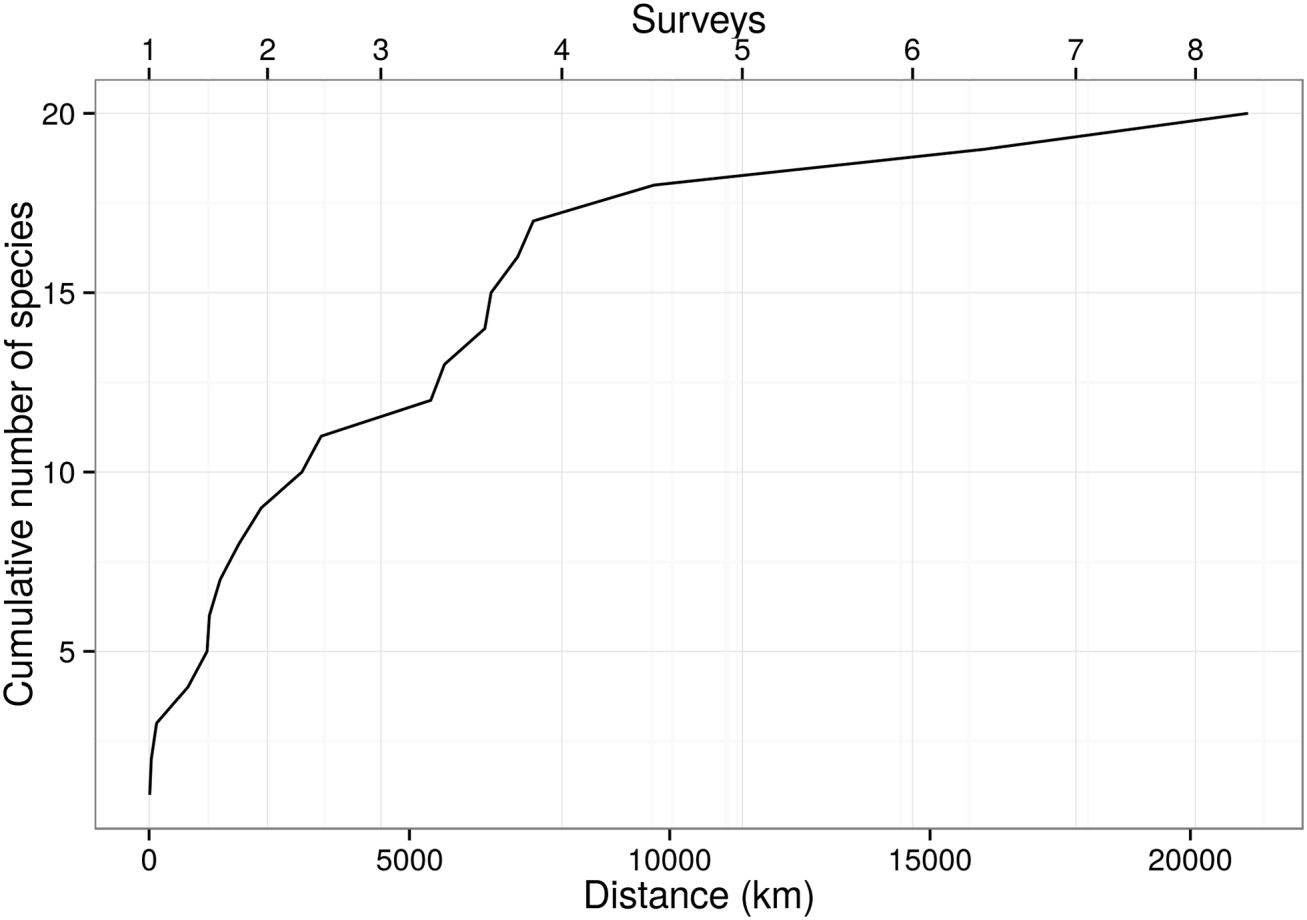
Discovery curve of species identified during the present study. Cumulative number of species recorded according to distance travelled (lower x-axis) and the surveys (upper x-axis).

**Fig 3:**
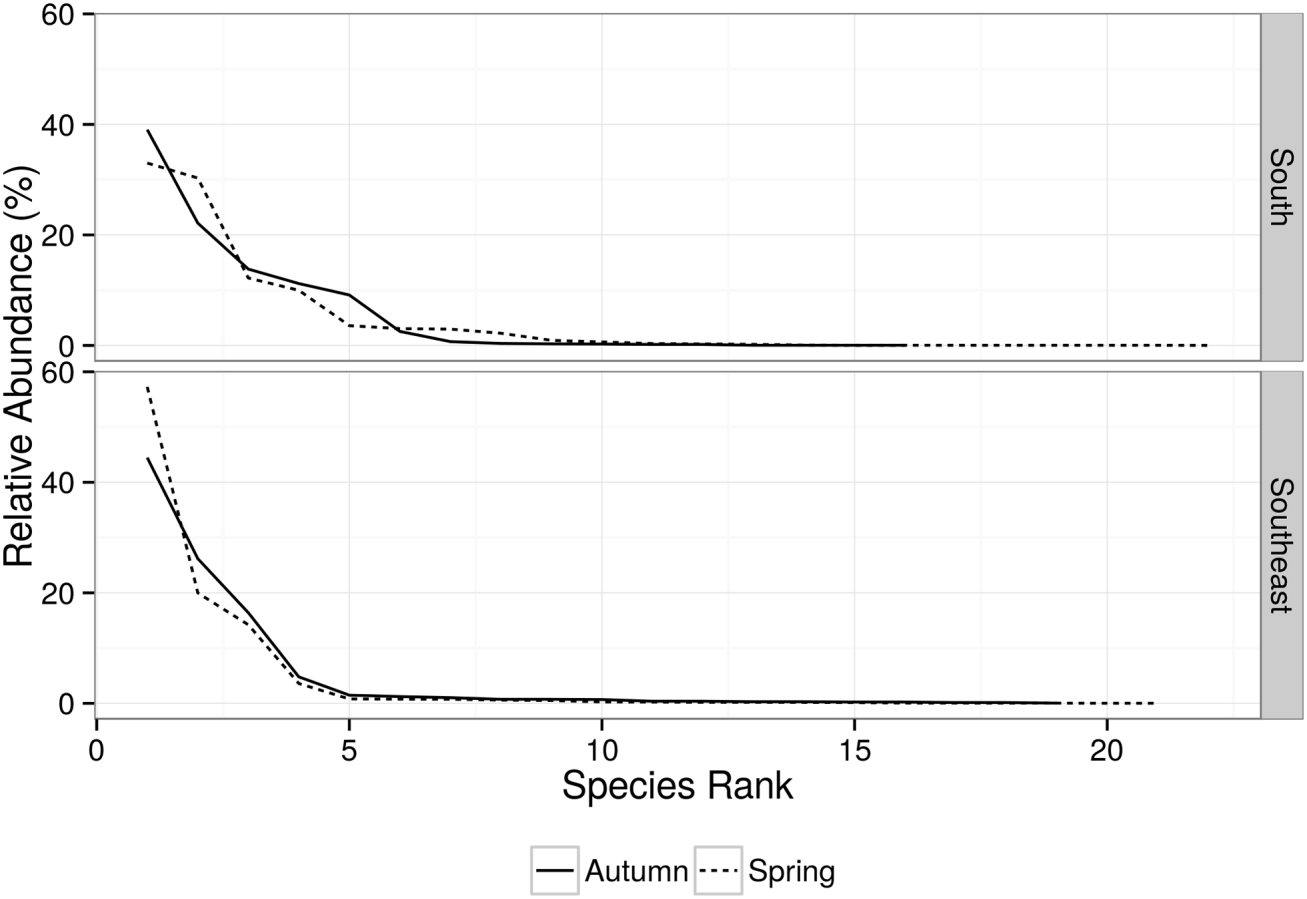
Whittaker plot of the relative species abundance by each area (South, Southeast) and season (Spring, Autumn). This plot considers the relative abundance as a proportion of the total number of individuals of each species in relation to the total number of individuals of all species.

Most (90%) of the 21 identified species were recorded during the first four surveys (Fig 2). Species richness (S) was similar between areas. It was also similar between seasons in the southeast area. However, when considering only the south area, S was almost twice as high in spring (15) as compared to autumn (9) (Table 2). Less than five species were dominant (*i*.*e*. presented relative abundance above 10% of overall number of individuals, considering all species) in each area and season (Fig 3). Beta-diversity (Harrison’s et al. modification of Whittaker index) ranged from 0.4 to 0.5, showing moderate turnover between seasons and areas (Table 4). The Morissita-Horn similarity index varied from 0.12 (spring—autumn) to 0.38 (south—southeast) and from 0.18 (spring—autumn in the southeast) to 0.59 (south—southeast during autumn). Intermediate values were found in other pair comparisons (Table 4). These values demonstrate that diversity is higher between seasons than between areas. It also suggests a higher diversity between seasons in the southeast and lower diversity between areas during autumn.

**Table 4:** Comparisons of beta-diversity indices between areas and seasons in the outer continental shelf and slope in the subtropical Southwestern Atlantic.

Areas and Seasons	Morisita-Horn	Whittaker-Harrison
South vs Southeast	0.376	-
Spring vs Fall	0.123	-
South: Spring vs Fall	0.186	0.4
Southeast: Spring vs Fall	0.178	0.5
Spring: South vs Southeast	0.372	0.4
Fall: South vs Southeast	0.587	0.5

vs, versus to represent comparisons between areas or seasons.

Mixed species assemblages were found in 5.2% of the cetacean sightings (n = 26). Half of the mixed groups had the presence of the common bottlenose dolphin (*Tursiops truncatus*). This species occurred in association with long-finned pilot whale (*Globicephala melas*, n = 10), Atlantic spotted dolphin (*Stenella frontalis*, n = 3), Risso’s dolphin (*Grampus griseus*, n = 4), false-killer whale (*Pseudorca crassidens*, n = 1) and sperm whale (n = 1). The other species found in mixed groups were spinner (*S*. *longirostris*) and pantropical spotted (*S*. *attenuata*) dolphins (n = 3), dwarf minke whale (*Balaenoptera acutorostrata*) and Atlantic spotted dolphin (n = 1), dwarf minke whale and an unidentified cetacean (n = 1), Antarctic minke (*B*. *bonaerensis*) and fin (*B*. *physalus*) whales (n = 1), and long-finned pilot whale with an unidentified whale (n = 1).

Overall cetacean ER did not differ between areas, however it was higher in spring compared to autumn (Tables 5 and 6). Furthermore, when considering the cetacean at group and species levels, ER varied between areas and seasons (Tables 5 and 6).

**Table 5:** Mean ER values for each area (South and Southeast) and season (Spring and Autumn).

Group categories/Species	Areas (S vs SE)	Seasons (Spr vs Aut)	South Area (Spr vs Aut)	Southeast area (Spr vs Aut)
Total Cetaceans	SE = 0.73(0.23) S = 0.96(0.37)	Spr = 1.25(0.44) Aut = 0.44(0.17)	Spr = 1.49(0.59) Aut = 0.44(0.16)	Spr = 1.01(0.28) Aut = 0.44(0.18)
Small delphinid	SE = 0.61(0.19) S = 0.71(0.29)	Spr = 1.05(0.26) Aut = 0.26(0.09)	Spr = 1.18(0.48) Aut = 0.23(0.13)	Spr = 0.93(0.27) Aut = 0.29(0.17)
Large delphinid	SE = 0.09(0.05) S = 0.21(0.07)	Spr = 0.15(0.07) Aut = 0.15(0.06)	Spr = 0.25(0.13) Aut = 0.17(0.05)	Spr = 0.06(0.02) Aut = 0.12(0.11)
Small whale	SE = 0.003(0.001) S = 0.002(0.001)	Spr = 0.003(0.001) Aut = 0.002(0.001)	Spr = 0.003(0.001) Aut = 0.001(0.0003)	Spr = 0.003(0.001) Aut = 0.003(0.001)
Large whale	SE = 0.007(0.002) S = 0.002(0.001)	Spr = 0.005(0.001) Aut = 0.004(0.002)	Spr = 0.002(0.001) Aut = 0.002(0.001)	Spr = 0.008(0.002) Aut = 0.007(0.005)
*Stenella frontalis*	SE = 0.21(0.08) S = 0.24(0.18)	Spr = 0.39(0.18) Aut = 0.05(0.02)	Spr = 0.44(0.36) Aut = 0.05(0.02)	Spr = 0.36(0.11) Aut = 0.05(0.04)
*Stenella longirostris*	SE = 0.35(0.13) S = 0.09(0.08)	Spr = 0.32(0.12) Aut = 0.12(0.09)	Spr = 0.17(0.15) Aut = 0	Spr = 0.47(0.17) Aut = 0.23(0.19)
Delphinus delphis*	S = 0.33(0.11)		Spr = 0.48(0.16) Aut = 0.19(0.11)	
*Tursiops truncatus*	SE = 0.03(0.01) S = 0.07(0.02)	Spr = 0.04(0.02) Aut = 0.06(0.02)	Spr = 0.05(0.03) Aut = 0.09(0.03)	Spr = 0.04(0.02) Aut = 0.02(0.02)
Globicephala melas**	SE = 0.005(0.003) S = 0.13(0.05)	Spr = 0.1(0.06) Aut = 0.03(0.02)	Spr = 0.19(0.09) Aut = 0.07(0.02)	Spr = 0.006Aut = 0.004
*Physeter macrocephalus*	SE = 0.01 (0.01) S = 0.04(0.01)	Spr = 0.026(0.01) Aut = 0.027(0.01)	Spr = 0.05(0.01) Aut = 0.03(0.02)	Spr = 0.01(0.003) Aut = 0.02(0.01)

ER, encounter rate; (), Standard error values;

* this species only occurred in the south area;

** species with one sighting and no standard error;

S,south area; SE, southeast; Spr, spring; Aut, autumn; vs, versus to represent comparisons between areas or seasons.

**Table 6:** ANOVA and post-hoc Tukey tests results of ER comparisons between areas south and southeast and seasons.

Group categories/Species	Areas (S vs SE)	Seasons (Spring vs Autumn)	South Area (Spring vs Autumn)	Southeast area (Spring vs Autumn)	Spring season (S vs SE)	Autumn season (S vs SE)
Total Cetaceans	p = 0.69	**p = 0.03**	p = 0.26	p = 0.56	p = 0.52	p = 0.99
Small delphinid	p = 0.88	**p = 0.01**	p = 0.09	p = 0.33	p = 0.98	p = 0.95
Large delphinid	p = 0.23	p = 0.99	p = 0.97	p = 0.97	p = 0.97	p = 0.57
Small whale	p = 0.39	p = 0.43	p = 0.89	p = 0.96	p = 0.95	p = 0.87
Large whale	p = 0.09	p = 0.40	p = 0.98	p = 0.82	p = 0.44	p = 0.74
*Stenella frontalis*	p = 0.72	**p = 0.01**	p = 0.62	p = 0.06	p = 0.94	p = 0.70
*Stenella longirostris*	p = 0.11	p = 0.26	p = 0.91	p = 0.75	p = 0.54	p = 0.74
Delphinus delphis*			p = 0.15			
*Tursiops truncatus*	p = 0.23	p = 0.78	p = 0.56	p = 0.34	p = 0.91	p = 0.12
*Globicephala melas*	**p = 0.01**	p = 0.66	p = 0.91	p = 0.99	p = 0.29	p = 0.10
*Physeter macrocephalus*	**p = 0.03**	p = 0.86	p = 0.92	p = 0.98	p = 0.17	p = 0.62

ER, encounter rate;

* this species only occurred in the south area, thus the only difference tested was between seasons;

vs, versus; S, south area; SE, southeast; p values in bold (p<0.05) represent statistically significant results.

### Cetacean distribution patterns

#### Sperm Whale

**Fig 4:**
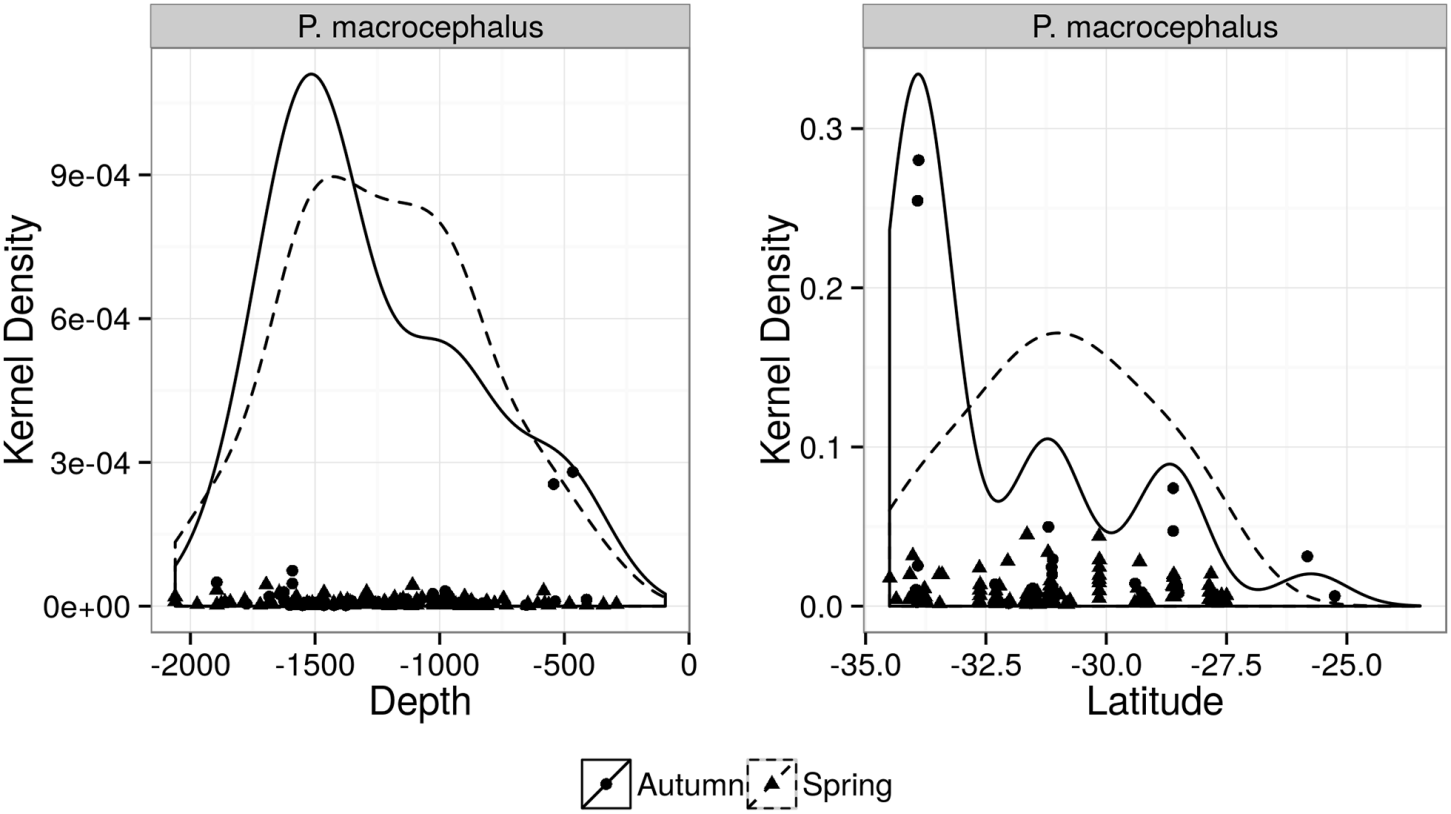
Kernel density of sperm whale (*Physeter macrocephalus*) distribution. Plots are according to depth (left) and latitude (right) during spring (dashed lines) and autumn (solid black lines) surveys.

The sperm whale was the most frequent species in the study area (n = 140), representing 27.8% of the all sightings (Table 2). Sperm whales occurred throughout the south area with only 19.2% (n = 27) of the sightings in the southeast. The ER was higher in the former at depths over 1000m (Tables 5 and 6, Fig 4). During autumn, fewer sightings (representing 25%, n = 35) of larger mean group sizes were registered (Table 2).

#### Small Delphinids

**Fig 5:**
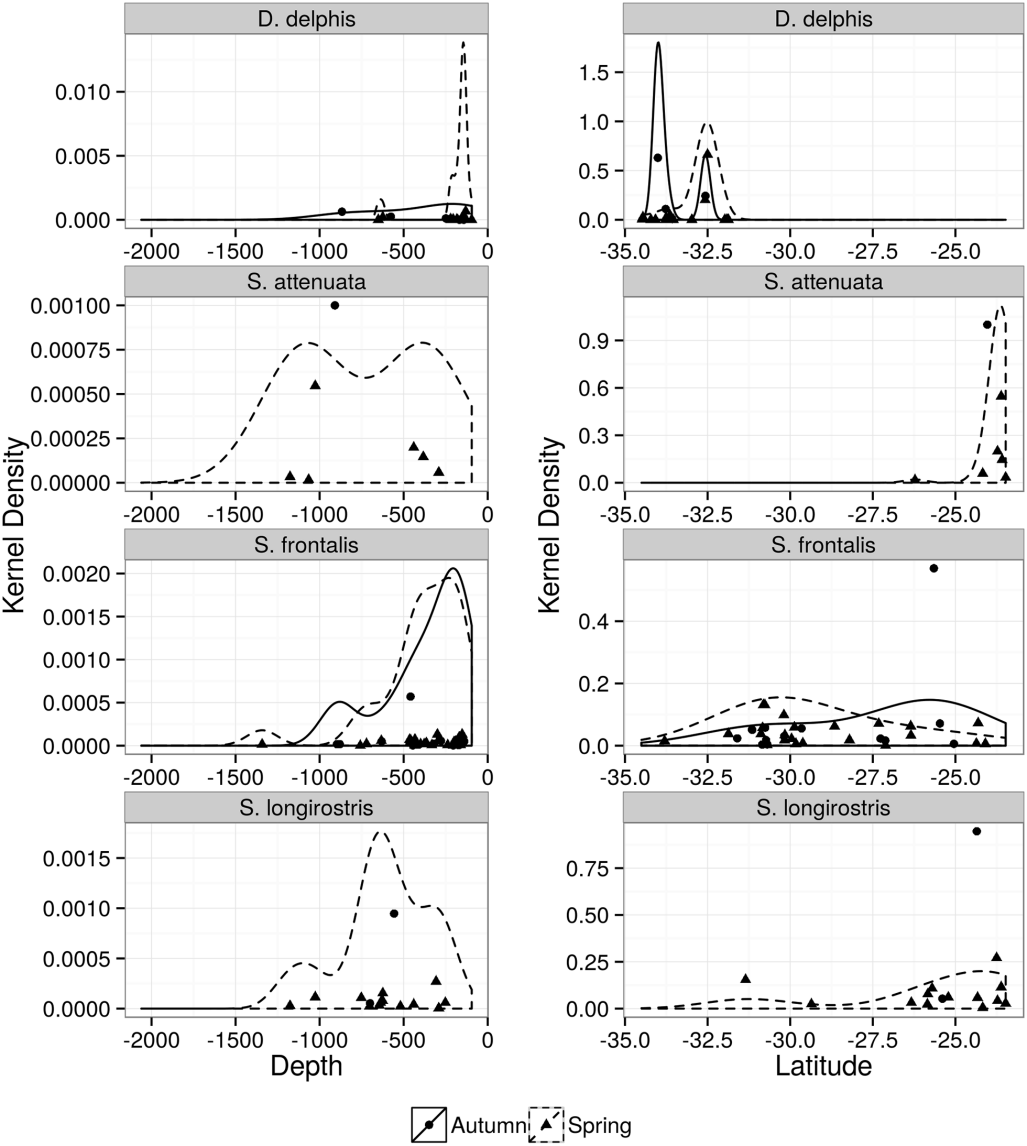
Kernel density distribution of Atlantic spotted dolphin (*Stenella frontalis*), short beaked common dolphin (*Delphinus delphis*), spinner dolphin (*Stenella longirostris*), pantropical spotted dolphin (*Stenella attenuata*). Plots are according to depth (left) and latitude (right) during spring (dashed lines) and autumn (solid black lines) surveys.

The small delphinids (n = 113 sightings) were evenly distributed along the study area with higher ER observed during spring (Tables 5 and 6, S1 Fig). The most frequent species was the Atlantic spotted dolphin (n = 40), followed by the common dolphin (n = 27), which occurred only in the south area. Higher densities of Atlantic spotted dolphin were observed in spring over depths around 250m and to the north of 31°S (Tables 5 and 6, Fig 5). However this difference in ER across seasons is apparently due to an increase in use of the southeast area during autumn. The short beaked common dolphin was only recorded to the south of 32°S. Although there was no difference in ER between seasons (Tables 5 and 6), larger mean group size were observed in autumn, mostly from the outer continental shelf to the upper slope (Table 2, Fig 5). Encounter rates of spinner dolphin (n = 15) and the few sightings of pantropical spotted (n = 7), which was observed only in the southeast, were also higher in spring and in deep waters over the continental slope (Tables 2, 5 and 6, Fig 5). The least frequent species, Clymene dolphin (*Stenella clymene*, n = 3), striped dolphin (*S*. *coeruleoalba*, n = 2), pygmy killer whale (*Feresa attenuata*, n = 1) and rough-toothed dolphin (*Steno bredanensis*, n = 1) were registered only during spring surveys (Table 2).

#### Large Delphinids

**Fig 6:**
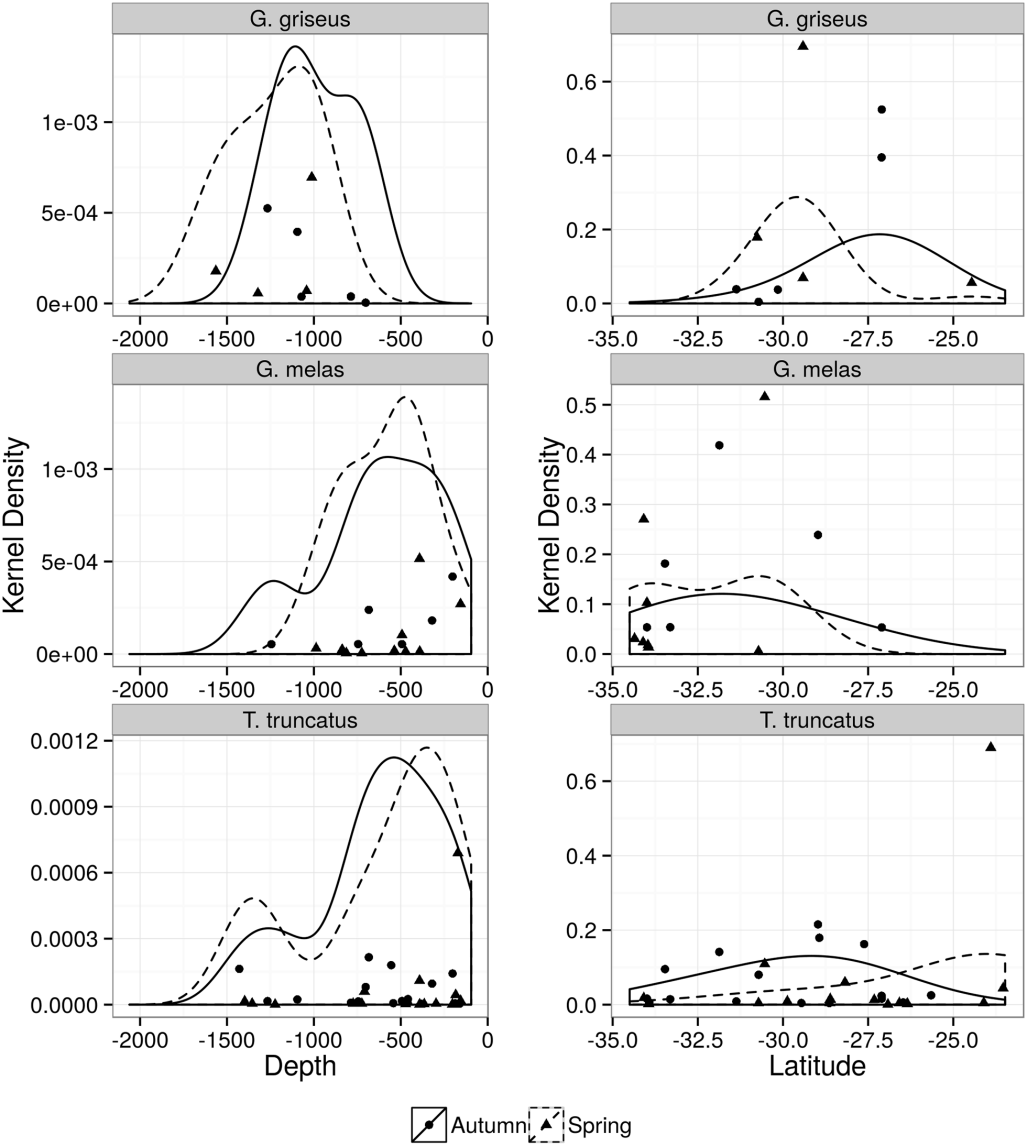
Kernel density distribution of Bottlenose dolphin (*Tursiops truncatus*), long-finned pilot whale (*Globicephala melas*), Risso’s dolphin (*Grampus griseus*). Plots are according to depth (left) and latitude (right) during spring (dashed lines) and autumn (solid black lines) surveys.

This group represented 13% (n = 67) of all sightings and occurred in both areas and seasons (Tables 2, 5 and 6; S2 Fig). The bottlenose dolphin was the most frequent (n = 33) species within this group and was distributed along the study area around depths of 500m (Fig 6). Although differences in ER were non-significant, higher densities shifted between seasons and areas. Densities were higher in the southeast during spring and in the south during autumn around the latitude of 29° (Tables 5 and 6, Fig 6). The second most frequent large delphinid, long-finned pilot whale (n = 17), presented higher densities in the south and during spring, in waters over the continental slope, similar to *bottlenose dolphins* (Tables 5 and 6, Fig 6). Risso’s dolphin (n = 9) was twice as frequent in the south as in the southeast, with higher densities beyond the 600m isobath (Table 2, Fig 6). Killer whale (*Orcinus orca*) was seen in five occasions, three in the south area during spring and two in the southeast area during autumn (Table 2). The only two sighting of false killer whale (*Pseudorca crassidens*) occurred in the southeast, one in each season (Table 2).

#### Small Whales

**Fig 7:**
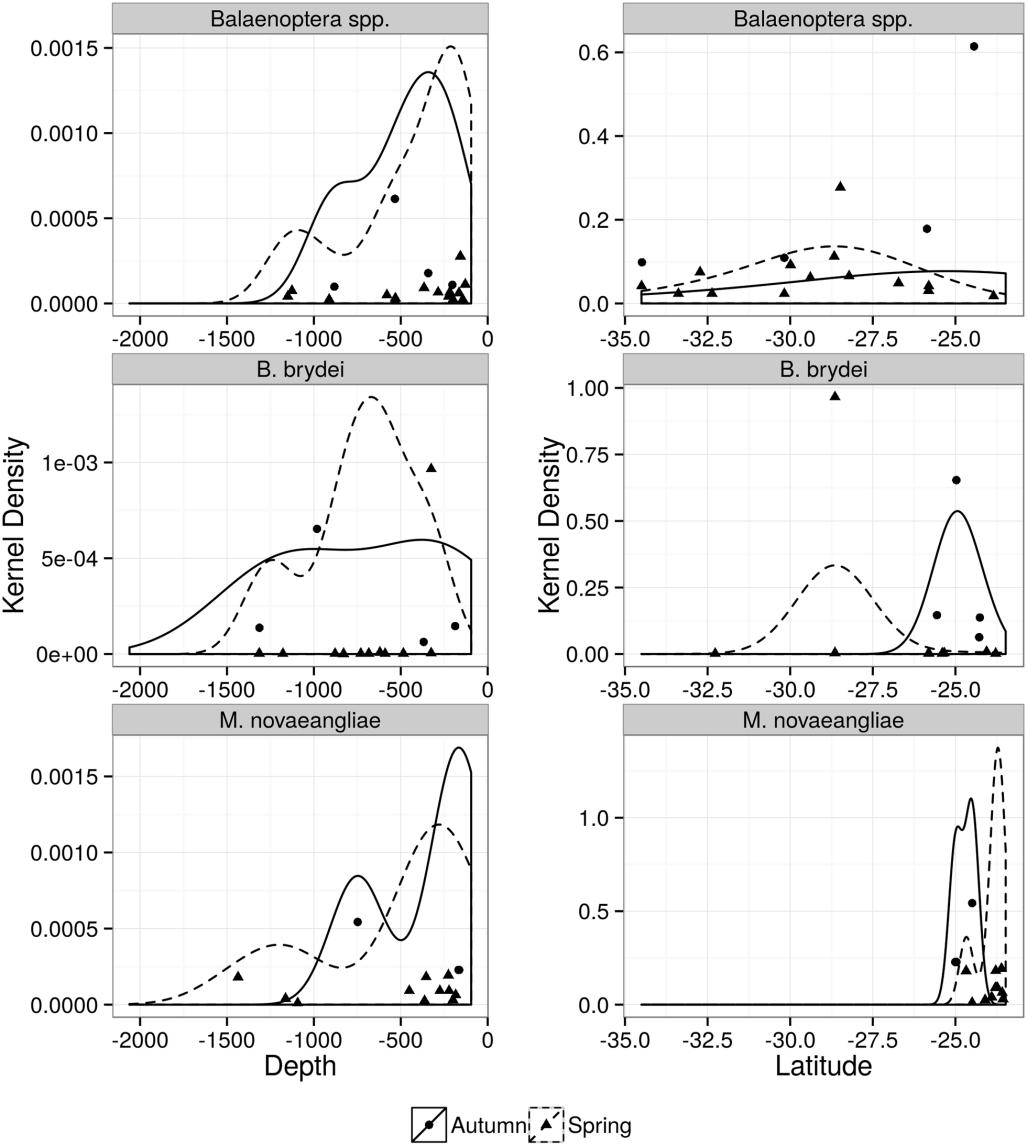
Kernel density distribution of unidentified minke whale (*Balaenoptera* spp.), Bryde’s whale (*Balaenoptera brydei*), humpback whale (*Megaptera novaeangliae*). Plots are according to depth (left) and latitude (right) during spring (dashed lines) and autumn (solid black lines) surveys.

This was the least sighted group (n = 30) and most sightings occurred in spring (n = 21). A large proportion of small whale sightings (48%) could not be identified to species level. Unidentified minke whale and the two minke whale species altogether accounted for 80% of the sightings within this group. There was no difference in the ER between areas and seasons (Tables 5 and 6), though sightings predominated in spring (S3 Fig). In general, higher densities of minke whales occurred around latitude 28°S during spring and 25°S in autumn, mainly in waters up to 500m deep (Fig 7).

#### Large Whales

This group accounted for 11.9% (n = 60) of all sightings (Table 2). No differences in ER were found between areas or seasons (Tables 5 and 6; S4 Fig). The two most frequently sighted large baleen whale species were Bryde’s (*Balaenoptera brydei*, *sensu* Pastene et al. 2015; n = 14) and the humpback (*Megaptera novaeangliae*, n = 14) whales (Table 2). Both species were mostly observed in the northern part of the southeast area during spring (Table 2, Fig 7). In the southeast, higher densities of Bryde’s whale were observed near the latitude of 25°S in both seasons (Fig 7). In the south, the species was only recorded twice in spring. Higher concentrations of Bryde’s whale occurred in waters between 500 and 1000m during spring (Fig 7). In autumn, sightings of humpback whale were composed of one individual, while in spring the species was mostly seen in pairs near the shelf break (Table 2, Fig 7). Fin whale was recorded only in three occasions during autumn surveys in both areas. Mean group size was larger compared to other large baleen whales (mean = 4.3, Table 2). Only one sei whale (*Balaenoptera borealis*) was registered in the south area in spring (Table 2).

## Discussion

### General Patterns of Cetacean Distribution

This study provides a comprehensive description of seasonal (autumn and spring) density and distribution for the most commonly encountered cetaceans in southern and southeastern Brazilian outer continental shelf and slope waters based on long term dedicated surveys. Despite differences in temporal and spatial coverage as well as methodological approaches (*e*.*g*. aerial vs ship-based surveys), species richness (n≥21) was similar to other productive areas worldwide such as the temperate waters of southern Australia (n≥15, [4]) and the California current system (n = 21, [44]), the tropical waters of Gulf of Mexico (n = 28, [6]), the eastern Pacific (n = 30, [45]) and the cold waters in the Pacific sub-Arctic gyres (n = 24, [46]). The fact that 90% of the species richness was recorded within the first four cruises indicates that the area was properly surveyed. Nevertheless, it is worth noting that our surveys were restricted to spring and autumn seasons, hence species richness for this area is possibly higher as typical tropical and cold-water cetaceans are most likely to occur in this area during summer and winter months, respectively. Furthermore, some cryptic species that are typically non-gregarious or solitary and inconspicuous such as beaked (Ziphiidae) as well as pygmy and dwarf sperm (Kogiidae) whales are known to occur in this area [18] but were not recorded in this study, except for one sighting of an unidentified ziphiid. Although the turnover of species between areas and seasons was moderate, diversity was higher between seasons than between areas. Diversity was also higher between seasons in the southeast and lower between areas during autumn. The moderate turnover suggests that there is no important variation in species composition when moving from one season or area to another. Higher turnover rates are likely to occur between seasons with extreme oceanographic variations, *i*.*e*. summer vs winter. In summer the entire area is dominated by the oligotrophic tropical water, therefore a dominance of tropical species is expected. During winter, while the southeast remains influenced by the tropical water, part of the south area receives inflow of cold and productive waters from Malvinas/Falkland current and subantarctic shelf water as well as the La Plata river plume, which together form the subtropical shelf front [24]. Thus, both tropical and cold-water species are likely to form the cetacean community in the study area. The influence of these productive waters extends towards the spring, at least in the south area. This is likely to explain both the higher diversity between seasons than between areas as well as a higher overall cetacean richness and density in spring, especially in the south area.

Cetacean densities were investigated through encounter rate index and were not corrected for animals missed on observation effort due to perception or availability bias (animals submerged, avoidance of or attraction to the vessel). Therefore the ER used as density index in this study reflects how these cetacean species used this area during the sampled years and does not show realistic estimates of densities values for this area. Overall, cetacean density was higher in spring compared to autumn surveys. Despite the fact that this pattern is mainly influenced by the most frequent and gregarious species (*e*.*g*. sperm whale, Atlantic spotted dolphin), this was expected as local productivity in subtropical/temperate waters of the Southwestern Atlantic [47] and hence prey availability is enhanced in spring. Although the outer continental shelf and slope of southeastern and southern Brazil are dominated by the oligotrophic tropical water carried out by the Brazil current, seasonal wind patterns control the behaviour of water masses and the occurrence of upwelling events affecting the local productivity, mainly in continental shelf waters [23,24,26]. The prevailing northeasterlies during spring and summer allow the southward tropical water and subtropical shelf water to reach the continental shelf and also promote the occurrence of local upwellings of the South Atlantic central water and transfer of continental derived materials to offshore areas [23]. On the other hand, the frequent southwesterlies during autumn and winter inhibit lateral circulation preventing offshore dispersion of nutrient rich coastal waters (especially in the south area) [23,24]. In the southeast, however, upwelling’s associated with cyclonic eddies enhance productivity on the outer continental shelf and slope [21,22]. The seasonal physical enrichment processes over the outer continental shelf and slope causes environmental discontinuities and are reflected in biological processes, such as patchiness of pelagic organisms. Small fishes of the family Myctophidae, squids and zooplankton (for baleen whales) are the most commonly prey species consumed by cetacean inhabiting offshore waters [46, 48, 49, 50]. Their distribution patterns are likely to affect the distribution and movements of their predators. Seasonal acoustic assessment studies carried out in the same area of this study have shown that lanternfish (*Maurolicus stehmanni*) and Myctophidae species are the most common mesopelagic fish with strong association with the tropical water over the continental slope [51]. The short fin squid (*Illex argentinus*) preys upon these small pelagic fish and is associated with cold waters. All these species play an important role in the pelagic food web of the outer continental shelf and slope of southern Brazil. They are preyed upon by a variety of fish [51,52], seabirds [53] and cetaceans [48, 53]. Densities of demersal fish species in the outer shelf and upper slope of southern Brazil are higher compared to the southeast area due to the influence of the southern nearby richer water masses [54].

### Spatial and Temporal Variation in Density of Cetacean Species

Higher densities of sperm whale were found in the deep waters over the continental slope of the south area in both seasons. The only few records in the southeast occurred in the southern end of that area. Furthermore, the species was more evenly distributed during spring while fewer groups composed of a larger mean number of individuals were observed during autumn. This species feed upon large squids [55] that are probably more associated to a demersal food web with different temporal pattern of productivity. Therefore the distribution pattern found in this study is possibly related to abundance of the short fin squids, one of the species’ main prey in this area [56, 57]. The short fin squid is associated with cold waters presenting high abundance during autumn mainly over the continental slope and a more sparse distribution with low abundances in late spring [57]. Probably this higher abundance of the short fin squid in autumn is a result of the enhanced productivity during spring as the development of all trophic levels between phytoplankton and large squids takes approximately four months [58]. The combination of oceanographic and topographic features appears to be relevant to sperm whales inhabiting other areas (*e*.*g*. in Mediterranean waters—[59, 60]). In the Gulf of California, sperm whales appear to change their distribution in response to a decline in abundance of squid species known to be their main prey [55]. In that area, during years of lower prey abundance sperm whales were evenly distributed compared with a year of prey’s high abundance, in which they were found in large aggregations.

The small delphinids were frequent in both areas along the outer continental shelf and slope, with higher densities observed in spring. Since the surveyed area is a transitional zone between oceanic and continental shelf water, the occurrence of representative species of both habitats was expected. Densities presented some latitudinal structure with varying overlap between species. Although similar patterns were described in previous studies [11, 13, 61, 62], our results add resolution on time and space scales of these species distribution over the outer continental shelf and slope.

The short beaked common dolphin only occurred in the south area and presented higher densities south of ~32°S and in waters shallower than 500m. According to Tavares *et al*. [62] the distribution of this species ranges from the outer continental shelf to upper slope (70–1500m), between southern Brazil and central Argentina, and is more restricted to shallower waters (18–70m) in southeastern Brazil. This pattern might explain the lack of sightings of short beaked common dolphin in southeast outer continental shelf and slope. The Atlantic spotted dolphin occurred throughout the study area, though higher density was observed north of 32°S, with only a few records south of this latitude. This latitude coincides with the northern limit of the short beaked common dolphin distribution during this study, suggesting some degree of habitat partitioning. Although the Atlantic spotted dolphin is found over the shallow continental shelf waters (20m) and slope (~1000m) in southeastern and southern Brazil [61, 63], areas south of 27°S and deeper than 200m are suggested to be less suitable for this species [11]. In our study, higher densities were observed beyond the shelf-break, which is consistent with previous studies that showed greater density of the Atlantic spotted dolphin at the outer shelf and slope [11, 61]. Despite methodological differences between studies, our findings show that waters beyond 200m presented high encounter rates for the Atlantic spotted dolphin both in spring and autumn as opposed to the model predictions by Amaral et al. [13].

Higher densities of spinner dolphin occurred in lower latitudes and beyond the shelf break. This species has been described to occur beyond the outer continental shelf in tropical waters of the Southwestern Atlantic Ocean [61, 13]; however, a few records exist south of 31°S, in spring. Sightings in autumn were rare and restricted to the southeast. This pattern is consistent with the preference of this species for a more tropical habitat and suggests that seasonal movements occur, possibly associated to foraging in more productive area in the south during spring. The few sightings of pantropical spotted and Clymene’s dolphins recorded in this study coincide with their more tropical distribution as proposed in previous studies [61,64]. Both species, however, occurred further south of their predicted suitable habitat as proposed by Amaral *et al*. [13]. These two species have similar habitat requirements and are reported in mixed associations in both the tropical Pacific and the Atlantic Oceans [13, 45]. Mixed groups involving small delphinids, as defined here, were registered only between pantropical spotted and spinner dolphins in the northern part of the southeast area and between Atlantic spotted and bottlenose dolphins. Although it has been proposed that the two species of spotted dolphins (*S*. *frontalis and S*. *attenuata*) are parapatric [61], the small overlap area in distribution was found to the north of the study area as previously suggested [13].

Rare sightings of pygmy killer whales, stripped and rough-toothed dolphins were consistent with their known distribution patterns [12]. The first is typically a tropical species, while the latter tends to occur over the continental shelf and *S*. *coeruleoalba* seems to be rare in the Southwestern Atlantic [12, 13].

Bottlenose dolphins was the most frequent species within the large delphinid group. The distribution of bottlenose dolphins is spread along the Southwestern Atlantic at both nearshore (coastal ecotype) and offshore (oceanic ecotype) waters [12]. The coastal ecotype concentrates in areas near river discharge, estuaries and bays of Argentina, Uruguay and southern Brazil [65]. The oceanic ecotype, on the other hand, seems to be widely distributed in tropical and subtropical deep waters along the outer continental shelf and beyond and in association with oceanic islands [12]. Nevertheless, small groups of the oceanic ecotype were shown to occur near shore in Rio de Janeiro (~23°S) during winter and spring and were inferred to be part of a larger offshore population using a wider geographic region [66]. In this study, only the oceanic ecotype was recorded. Higher densities of bottlenose dolphins were observed between 28°S and 30°S, from the outer continental shelf to the upper slope, in both seasons. Bottlenose dolphin was also the species mostly seen (53% of its records) in multispecific associations. These associations occurred with five species, though no more than two species were involved each time. Most of the associations were observed with long-finned pilot whale and Risso’s dolphin and occurred over the continental slope of the south area. The reasons of these multispecific associations are unknown but are likely related to foraging or predator avoidance [67].

Sightings of long-finned pilot whale occurred mainly south of 30°S and depths between 500 and 1000m, similarly to previous reports [15]. This depth range is comparable to that of bottlenose dolphin and might explain the relatively frequent occurrence of mixed groups of these two species reported here. Despite the limited number of records of Risso’s dolphin during this study, the higher densities between 28°S and 30°S in offshore waters is in accordance with the preference for subtropical/temperate waters of continental shelf and slope described as the species distribution pattern worldwide [60, 68].

Although sightings of killer whale were rare, this species was shown to frequently depredate the catch of longline fisheries near the shelf break and beyond in this region [69, 70].

Most sightings of small whales included minke whales that could not be identified to species level due to the difficulty in assessing differences in colour patterns between the two species at long distances. Minke whales were present in both seasons with a higher frequency and density in spring. This pattern coincides with sighting and stranding records of these species in this region [18, 71, 72]. The high occurrence of these species in spring is probably related to the use of the outer continental shelf and upper slope as part of the migration pathway from tropical and subtropical breeding grounds off Brazil towards feeding grounds. It has been suggested that Antartic minke whale occupies deeper waters beyond 200m isobath while dwarf minke whale is distributed primarily in shallower waters over the continental shelf [17, 71, 72, 73]. Although our survey area does not cover the mid and inner continental shelf, the high number of sightings (both species) beyond the shelf break and the fact that few confirmed sightings of dwarf minke whale occurred in deeper waters (mean depth of 553m –Table 2) suggest that both species are commonly distributed along the shelf break and slope. The few sightings of both minke whale species in autumn are evidence that some individuals do not migrate to sub-Antarctic and Antarctic feeding grounds or are arriving earlier at the breeding grounds. This has been suggested in previous studies based on summer and autumn records in stranding, sighting and whaling data [18, 71, 72] and indicates that a small fraction of the population of both species can be maintained by local productivity in subtropical waters.

Higher densities of humpback and Bryde’s whales were observed in the southeast area and this is to opposite examples of large whale behaviour. Bryde’s whale has been referred as *Balaenoptera edeni* until recently when a phylogenetic analysis confirmed that in South America the species is *B*. *brydei* [74]. The Bryde whale occupied a wide area from the outer continental shelf to the slope; however, higher densities were observed over the upper slope (~750m isobath), during spring and at lower latitudes. This species has been reported to occur year round in coastal areas of southeast Brazil [75]. Bryde’s whale does not perform long-distance seasonal movements as other balaenopteriids, though, despite the limited number of sightings, there is evidence that the species uses the south area during spring. The low number of sighting in autumn also corroborates with other studies which proposed that this species perform movements to other feeding grounds, including coastal areas not surveyed in this study [72, 75]. Changes in the species distribution have been related to prey availability [76, 77].

The humpback whale, on the other hand, is well known to perform long-distance seasonal migrations between winter/breeding and summer/feeding areas in low and high latitudes [78]. During winter and spring, humpback whale occurs from northeastern Brazil (~5°S) to Rio de Janeiro state (~23°S) [73]). Therefore, the higher densities observed in spring surveys and only in the southeast area were expected, particularly because the northern range of this study area is used by humpback whales during their southbound spring migration [79]. At the time of the surveys (approaching mid spring), the whales were starting their southward migration to feeding grounds in sub-Antarctic waters. The lack of sightings in the south area coincides with the species’ far offshore migration pathway [79, 80].

The low number of sightings of fin and sei whales is probably related to their relatively lower abundances and/or further offshore distribution in lower latitudes of the Southwestern Atlantic [81, 82, 83].

The results presented here strongly emphasizes on the importance of the outer continental shelf and slope to a diverse community of cetaceans occurring in the subtropical Southwestern Atlantic. This work contributed to improve in description of the distribution pattern at both temporal and spatial scales for the most frequent cetacean species. Areas of higher diversity and density that are persistent in time are strong candidates to be declared ecologically and biologically significant areas and to receive special attention for conservation in situations of conflict with human activities.

## Supporting Information

S1 Fig Encounter rate (ER) distribution of small delphinids during spring (left) and autumn (right) surveys in the south and southeastern Brazil. Acronyms represent the Braziliam states of Rio Grande do Sul (RS); Santa Catarina (SC); Paraná (PR); São Paulo (SP) and Rio de Janeiro (RJ). Dashed line is the limit between south and southeast areas. Solid grey lines are 200m, 1500m, 2000m isobaths.
(TIF)

S2 Fig Encounter rate (ER) distribution of large delphinids during spring (A) and autumn (B) surveys in the south and southeastern Brazil. Acronyms represent the Brazilian states of Rio Grande do Sul (RS); Santa Catarina (SC); Paraná (PR); São Paulo (SP) and Rio de Janeiro (RJ). Dashed line is the limit between south and southeast areas. Solid grey lines are 200m, 1500m, 2000m isobaths.
(TIF)

S3 Fig Encounter rate (ER) distribution of small whale during spring (left) and autumn (right) surveys in the south and southeastern Brazil. Acronyms represent the Brazilian states of Rio Grande do Sul (RS); Santa Catarina (SC); Paraná (PR); São Paulo (SP) and Rio de Janeiro (RJ). Dashed line is the limit between south and southeast areas. Solid grey lines are 200m, 1500m, 2000m isobaths.
(TIF)

S4 Fig Encounter rate (ER) distribution of large whales during spring (left) and autumn (right) surveys. Acronyms represent the Brazilian states of Rio Grande do Sul (RS); Santa Catarina (SC); Paraná (PR); São Paulo (SP) and Rio de Janeiro (RJ). Dashed line is the limit between south and southeast areas. Solid grey lines are 200m, 1500m, 2000m isobaths.
(TIF)
