# Supplementary figures and images for: Diversity and Distribution Patterns of Cetaceans in the Subtropical Southwestern Atlantic Outer Continental Shelf and Slope
Source: PLoS One. 2016 May 31;11(5):e0155841. doi: 10.1371/journal.pone.0155841 (PMC4887039; doi:10.1371/journal.pone.0155841)

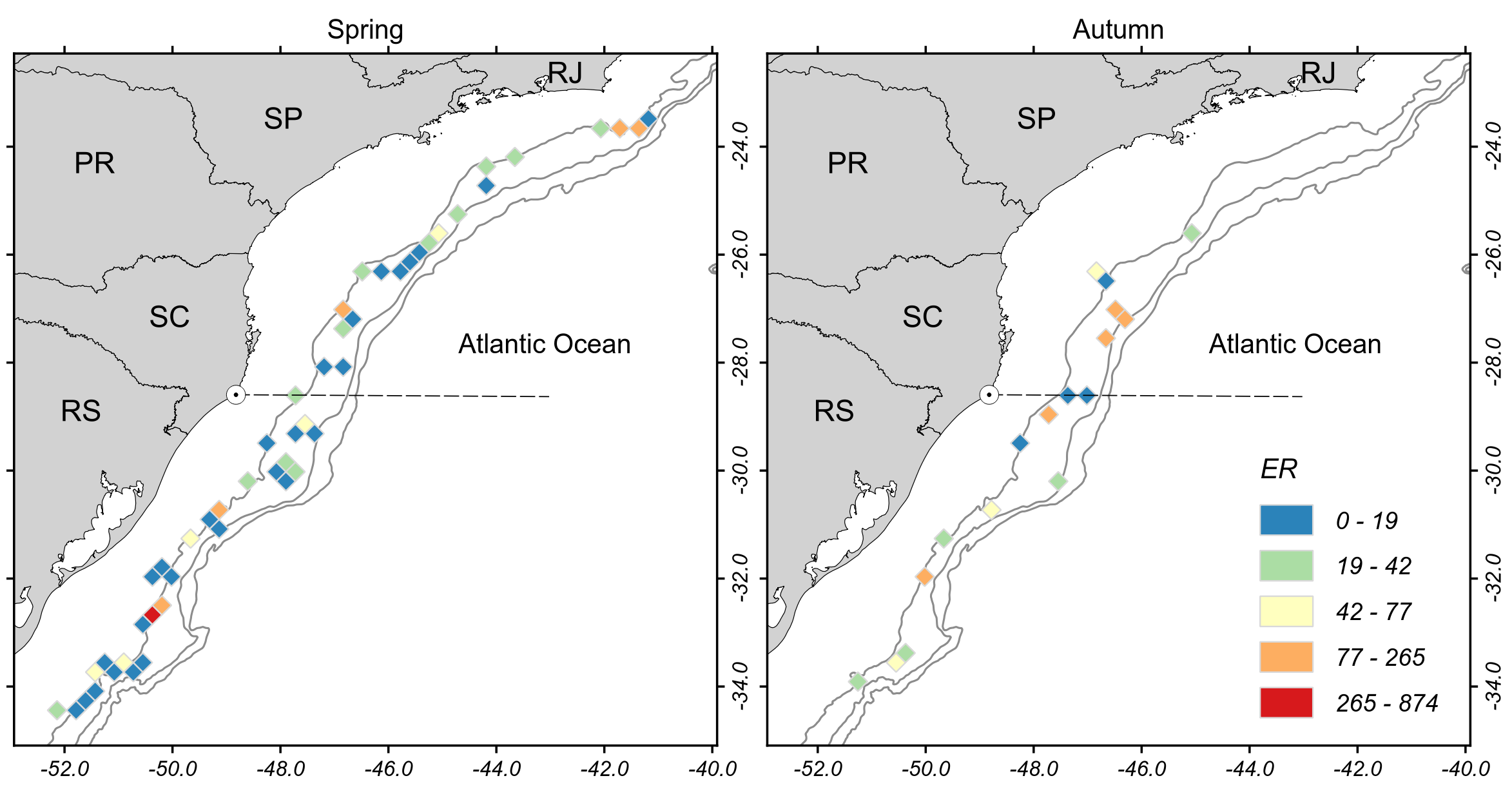

Supplement: S1 Fig — Acronyms represent the Braziliam states of Rio Grande do Sul (RS); Santa Catarina (SC); Paraná (PR); São Paulo (SP) and Rio de Janeiro (RJ). Dashed line is the limit between south and southeast areas. Solid grey lines are 200m, 1500m, 2000m isobaths. (TIF) [file pone.0155841.s001.tif]

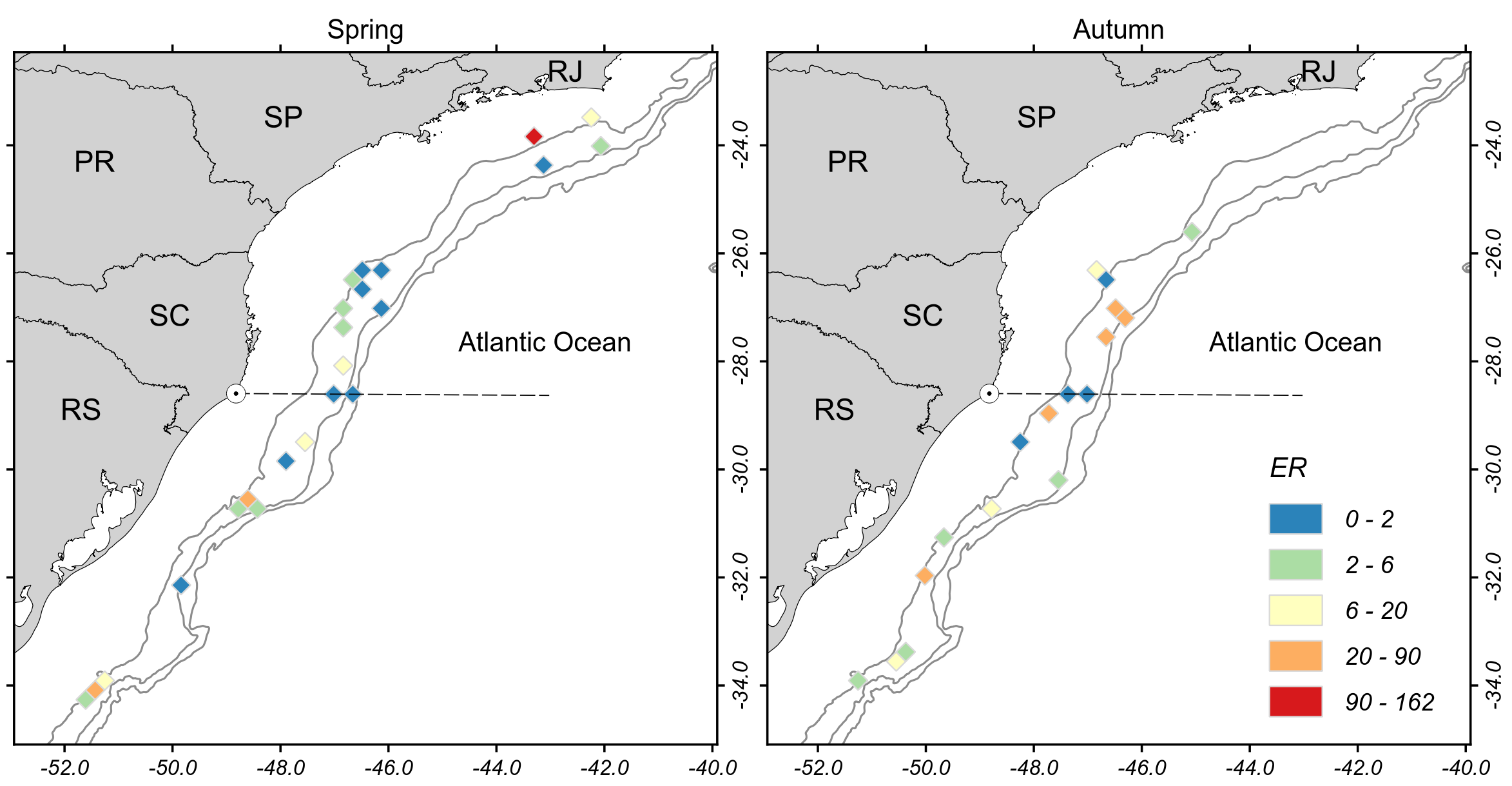

Supplement: S2 Fig — Acronyms represent the Brazilian states of Rio Grande do Sul (RS); Santa Catarina (SC); Paraná (PR); São Paulo (SP) and Rio de Janeiro (RJ). Dashed line is the limit between south and southeast areas. Solid grey lines are 200m, 1500m, 2000m isobaths. (TIF) [file pone.0155841.s002.tif]

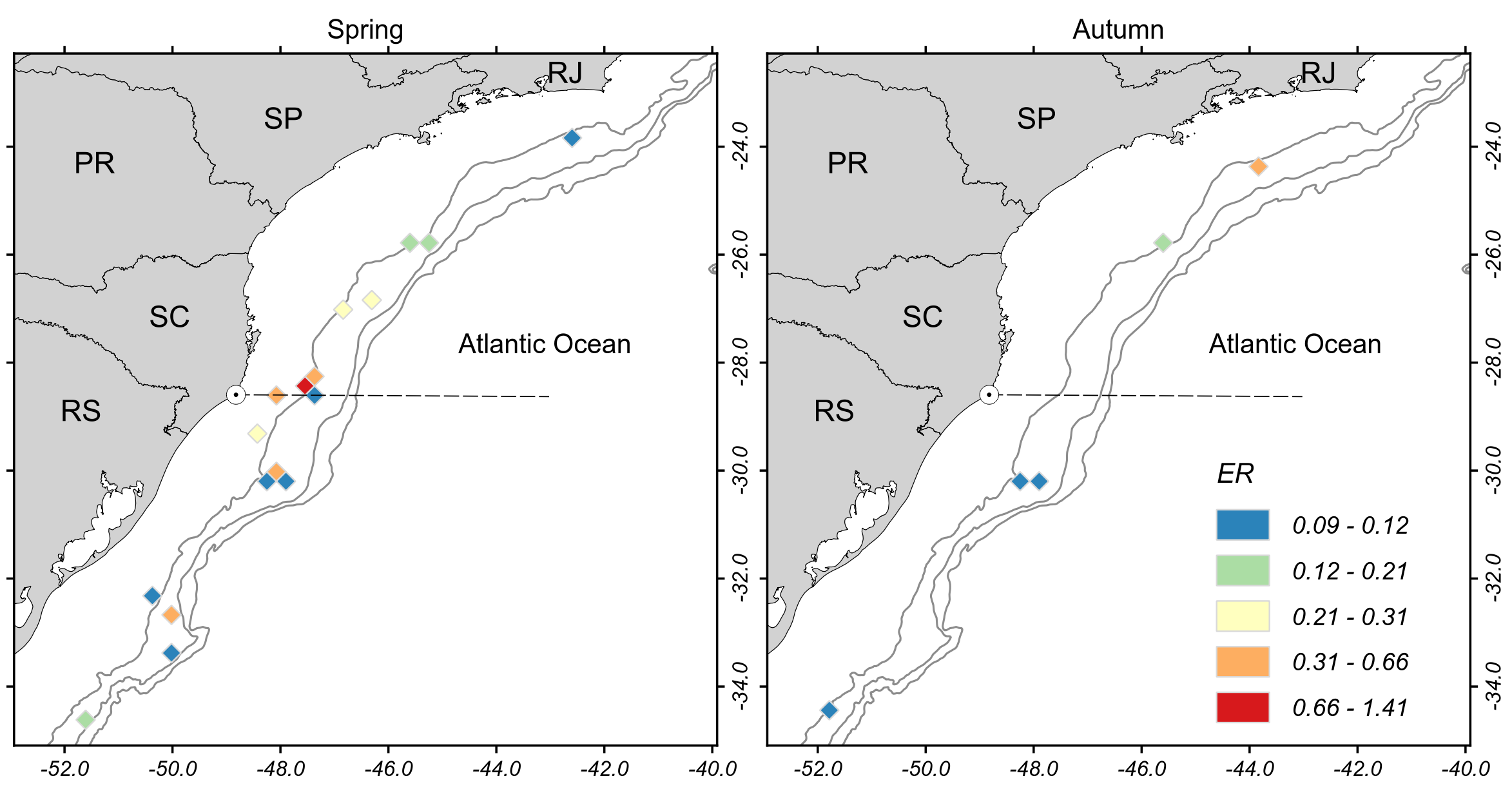

Supplement: S3 Fig — Acronyms represent the Brazilian states of Rio Grande do Sul (RS); Santa Catarina (SC); Paraná (PR); São Paulo (SP) and Rio de Janeiro (RJ). Dashed line is the limit between south and southeast areas. Solid grey lines are 200m, 1500m, 2000m isobaths. (TIF) [file pone.0155841.s003.tif]

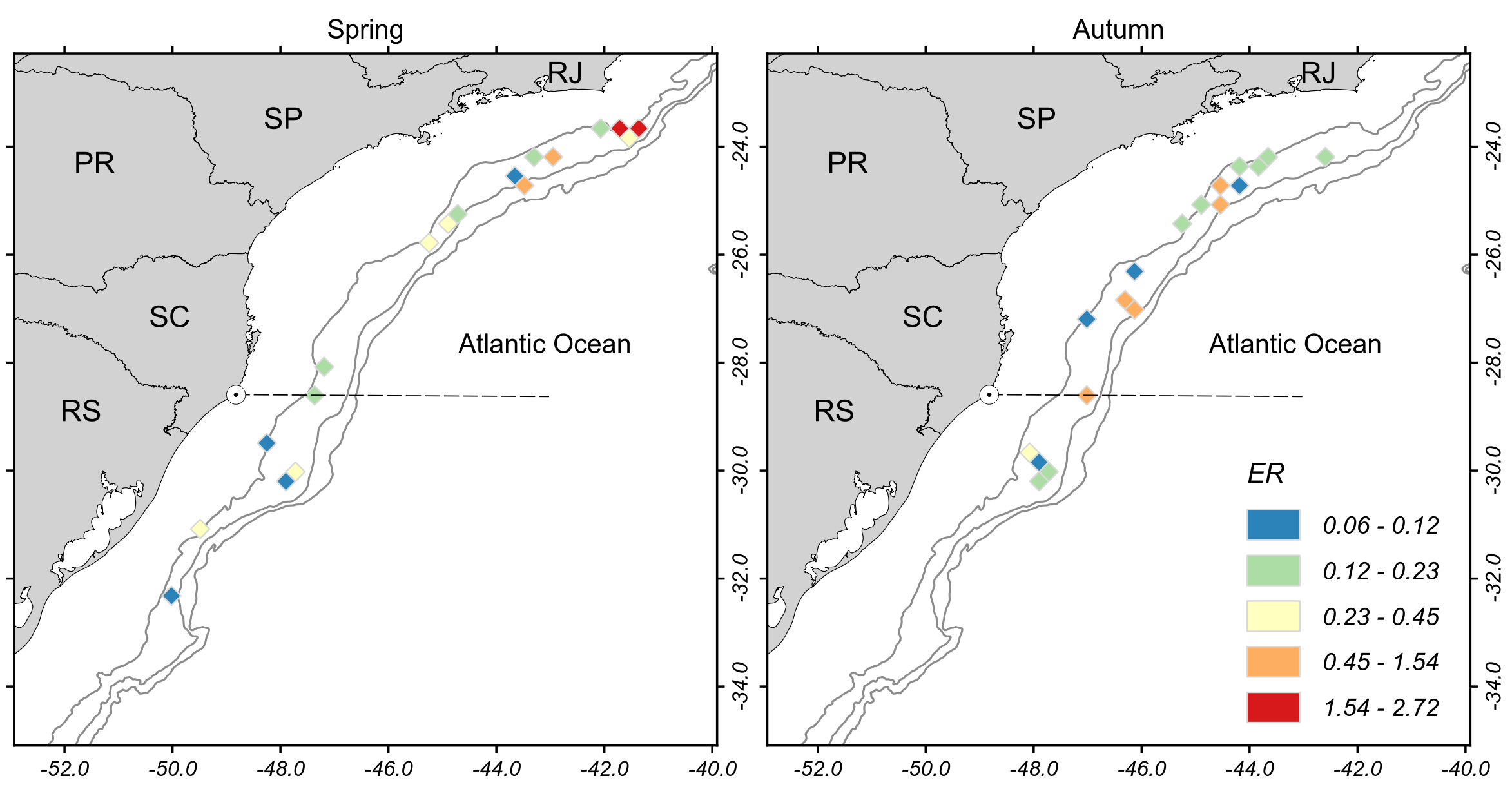

Supplement: S4 Fig — Acronyms represent the Brazilian states of Rio Grande do Sul (RS); Santa Catarina (SC); Paraná (PR); São Paulo (SP) and Rio de Janeiro (RJ). Dashed line is the limit between south and southeast areas. Solid grey lines are 200m, 1500m, 2000m isobaths. (TIF) [file pone.0155841.s004.tif]
